# B Cell Siglecs–News on Signaling and Its Interplay With Ligand Binding

**DOI:** 10.3389/fimmu.2018.02820

**Published:** 2018-12-03

**Authors:** Sarah J. Meyer, Alexandra T. Linder, Carolin Brandl, Lars Nitschke

**Affiliations:** Division of Genetics, Department of Biology, University of Erlangen, Erlangen, Germany

**Keywords:** CD22, B lymphocytes, inhibitory receptors, sialic acid, Siglec

## Abstract

CD22 and Siglec-G are members of the Siglec family. Both are inhibitory co-receptors on the surface of B cells and inhibit B-cell receptor induced signaling, characterized by inhibition of the calcium mobilization and cellular activation. CD22 functions predominantly as an inhibitor on conventional B cells, while Siglec-G is an important inhibitor on the B1a-cell subset. These two B-cell Siglecs do not only inhibit initial signaling, but also have an important function in preventing autoimmunity, as double deficient mice develop a lupus-like phenotype with age. Siglecs are characterized by their conserved ability to bind terminal sialic acid of glycans on the cell surface, which is important to regulate the inhibitory role of Siglecs. While CD22 binds α2,6-linked sialic acids, Siglec-G can bind both α2,6-linked and α2,3-linked sialic acids. Interestingly, ligand binding is differentially regulating the ability of CD22 and Siglec-G to control B-cell activation. Within the last years, quite a few studies focused on the different functions of B-cell Siglecs and the interplay of ligand binding and signal inhibition. This review summarizes the role of CD22 and Siglec-G in regulating B-cell receptor signaling, membrane distribution with the importance of ligand binding, preventing autoimmunity and the role of CD22 beyond the naïve B-cell stage. Additionally, this review article features the long time discussed interaction between CD45 and CD22 with highlighting recent data, as well as the interplay between CD22 and Galectin-9 and its influence on B-cell receptor signaling. Moreover, therapeutical approaches targeting human CD22 will be elucidated.

## B cell siglecs – controlling B-cell receptor signaling

The activation of the B-cell receptor (BCR) signaling pathway is an important step in starting a B-cell response. However, as essential as initiating this process, it is necessary to limit signaling and signaling strength in order to prevent hyperactivity of the B cell. Lack of appropriate BCR inhibition is often associated with dysregulation of the B-cell immune response, which can lead to autoimmunity. Therefore, B-cell activation needs to be tightly balanced, which is ensured by different activating and inhibitory receptors on the B-cell surface. Among those, proteins of the Sialic acid binding immunoglobulin like lectin (Siglec) family play an important role in regulating BCR signaling (1). The Siglec family contains a set of transmembrane proteins that share certain structural similarities. They are characterized by an extracellular domain, consisting of various numbers of Immunoglobulin (Ig) domains with a conserved N-terminal V-set Ig ligand binding domain. Moreover, they have a transmembrane region and a cytoplasmic tail with signaling motifs, in most cases immunoreceptor tyrosine-based inhibitory motifs (ITIM). Sialic acids, which terminate carbohydrate structures of glycans, are the ligands of Siglec proteins. Different Siglecs show preferential binding to sialic acids in different linkages (2).

B cells express two Siglecs on their cell surface, named CD22 (Siglec-2) (3) and Siglec-G (4, 5), which are known to inhibit BCR signaling. Both carry ITIMs within their cytoplasmic tail and recruit the tyrosine phosphatase SHP-1 that inhibits cell signaling (5–9). CD22 expression is B cell restricted (3) and has its main function on conventional B cells (also called B2-cells) as shown by different groups analysing CD22 knockout mice (10–13). In contrast, Siglec-G, which is not only expressed on B cells, but also on dendritic cells and eosinophils (4, 5), inhibits BCR signaling on the B1a cell population (14). Both Siglecs show the family typical binding of sialic acids. CD22 binds α2,6-linked sialic acids (15, 16), while Siglec-G can bind both, α2,6- and α2,3-linked sialic acids (17). Ligand binding can occur in *cis*, which means to sialic acids on the same cell surface, or in *trans* to sialic acids expressed on other cells (2, 18). Interestingly the lack of CD22 leads to a pre-activated B cell phenotype with a higher calcium mobilization, but this does not cause autoimmunity on a pure C57BL/6 background (10, 12, 13), while autoimmunity has been observed on a mixed 129 x C57BL/6 background (11). Siglec-G deficient mice show an expanded B1a cell population with higher calcium influx upon BCR stimulation. In this strain, age-related autoimmunity occurs on C57BL/6 background (19). Furthermore, Siglec-G deficiency accelerates the onset of disease in autoimmune mouse models, for example in collagen-induced arthritis or lupus-prone MRL/lpr mice (20). However, a double deficient mouse, lacking both Siglec-G and CD22, develops systemic lupus-like autoimmune disease with age, demonstrating a partly redundant function of these two Siglecs on B cells (21). This clearly shows the importance of Siglecs in regulating B-cell activation in order to prevent hyperactivity of B cells. This review summarizes interesting new findings about the physiological role of these two B cell Siglecs.

## CD22 – new insights on its signaling function

The signaling function of CD22 has been investigated for several years and a lot of studies characterized the 6 cytoplasmic tyrosines, their different binding partners and downstream signaling (7, 8, 22, 23). More recently, two different knockin mice were generated in order to dissect CD22 ligand binding and cytoplasmic signaling function (24). The CD22-R130E mutant mouse has a defect in the ligand binding domain, as the conserved arginine at position 130 has been replaced by a glutamic acid. As a result of this mutation, CD22 is not able to bind its ligand α2,6-linked sialic acid anymore, however, the intracellular tail is still intact. The other mouse strain, named CD22-Y2,5,6F, carries point mutations at the highly-conserved cytoplasmic tyrosines 2 (Y783), 5 (Y843), and 6 (Y863), while showing unchanged ligand binding. Each of these tyrosines is located within one of the three ITIMs and is replaced by a phenylalanine in this knockin mouse. This work nicely showed a reduced CD22 phosphorylation in these mutant mice. Furthermore, it was confirmed that the tyrosine phosphatase SHP-1, which has been shown to bind to phosphorylated ITIMs of CD22 upon BCR stimulation (7), is not binding to CD22-Y2,5,6F anymore (24). By comparing ligand binding deficient mice to ITIM mutant mice, Müller et al. (24) were able to assign the different phenotypes of the CD22 knockout mouse to the ligand binding or the signaling domain of CD22. Consequences of a defective signaling are a reduced number of mature recirculating B cells in the bone marrow. This reduction was explained with a higher turnover of mature B cells, as measured by BrdU incorporation and apoptosis rate. Additionally they analyzed calcium mobilization after BCR stimulation. Like expected, they could show an increase in calcium mobilization compared to wildtype (WT) mice, confirming that the phosphorylation of CD22 ITIMs are crucial to inhibit calcium signaling in B cells (24).

**Figure 1:**
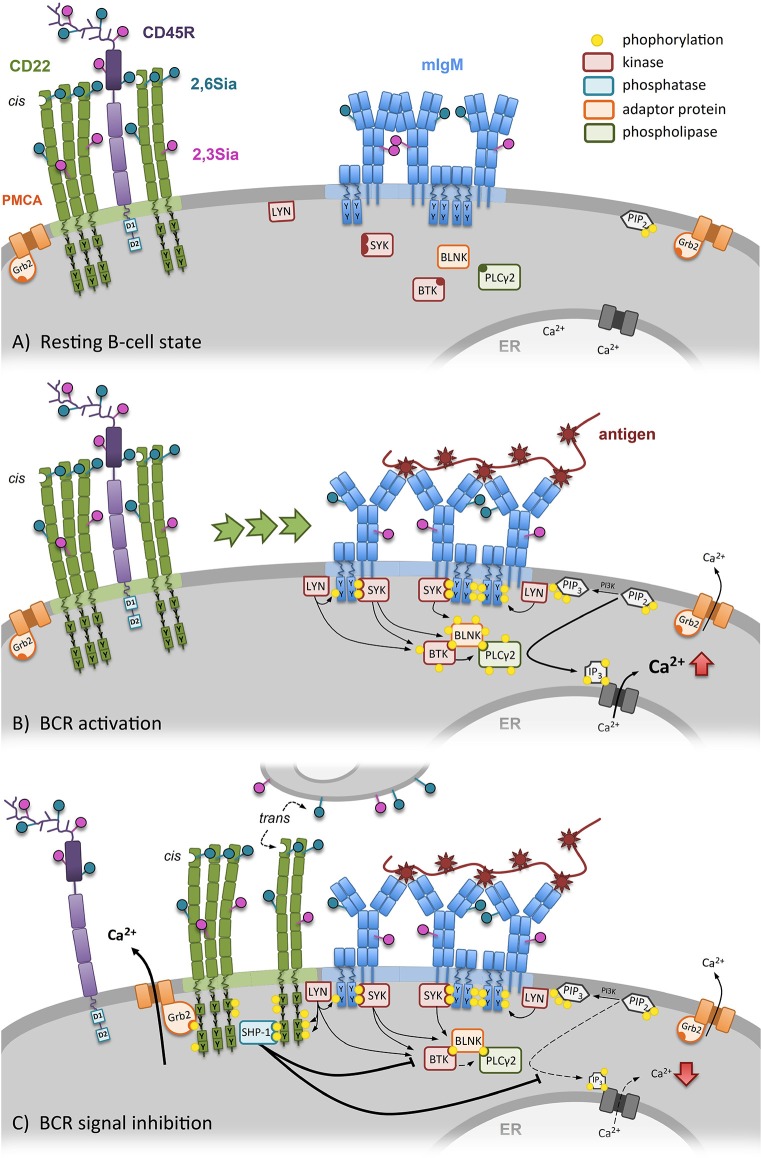
CD22 dependent regulation of the B-cell receptor (BCR) signal. **(A)** In resting B cells the conformation of the BCR is closed and CD22 is forming homooligomers (*cis*-interaction) distinct from the BCR. **(B)** Specific antigen binding induces conformational opening of the BCR, followed by activation and phosphorylation of ITAMs of the Igα/Igβ-complex. Increased SYK recruitment and activation of the BTK-BLNK-PLCγ2-complex leads to more Ca^2+^-release out of the endoplasmatic reticulum. Additionally, CD22 clusters are recruited to the BCR. **(C)** After BCR activation, CD22 recruitment inhibits the BCR signaling. Additionally, CD22 can also be recruited to the BCR by bind its ligands on other cells (*trans*-interaction). Due to vicinity of CD22 to BCR, ITIMs of CD22 get phosphorylated by LYN. SHP-1 binds to CD22 and inhibits further Ca^2+^ release. In addition, through the formed CD22-Grb2-PMCA complex, Ca^2+^ is transported out of the cell into the extracellular space by PMCA.

It has been reported that CD22 interacts with and potentiate the activity of the plasma membrane calcium ATPase PMCA (a calcium pump) and is therefore important to terminate calcium responses in the B cell after BCR stimulation (25). A nice study focused in more detail on the CD22 dependent activation of PMCA and dissected the tyrosines involved in this pathway. They reported a role of the CD22 tail tyrosine Y4, but not Y2,5 or 6 in the association with PMCA (26). The pY4 within the YENV motif has been known since the late 90s to bind the adaptor protein Grb2 (8, 27, 28). However, a physiological role has been missing so far. Now, Chen et al. (26) demonstrated that the CD22-PMCA association is Grb2 dependent, which in turn is already bound to PMCA in the steady state (26). Additional support comes from studies with B-cell-specific Grb2-deficient mice. These mice show an elevated calcium mobilization in mature and immature B cells (29, 30). To conclude, CD22 mediates regulation of calcium signaling through two different signaling pathways via two associated signaling proteins (SHP-1 and Grb-2), binding to distinct phosphorylated tyrosines of its intracellular signaling domain (Figure 1).

## Siglec-G – inhibitory function on the B1-cell subset

Siglec-G and its human ortholog named Siglec-10 belong to the CD33-related Siglecs. Both, murine and human version, carry one ITIM and one ITIM-like domain within the cytoplasmic tail and additionally have a Grb2 binding site (2). The signaling cascade of Siglec-G has not been studied extensively, however SHP-1 and SHP-2 have been identified as phosphorylation-dependent binding partner of human Siglec-10. In this study, the cytosolic domain of Siglec-10 GST fusion proteins were phosphorylated by tyrosine kinases. Afterwards they were incubated with cell lysates, followed by a GST pulldown assay and anti-SHP-1 or SHP-2 western blot. Using single mutations of the relevant tyrosines, it was shown that Y667 is the key tyrosine that needs to be phosphorylated in order to recruit SHP-1 and is also partially important for SHP-2 binding (9).

**Figure 2:**
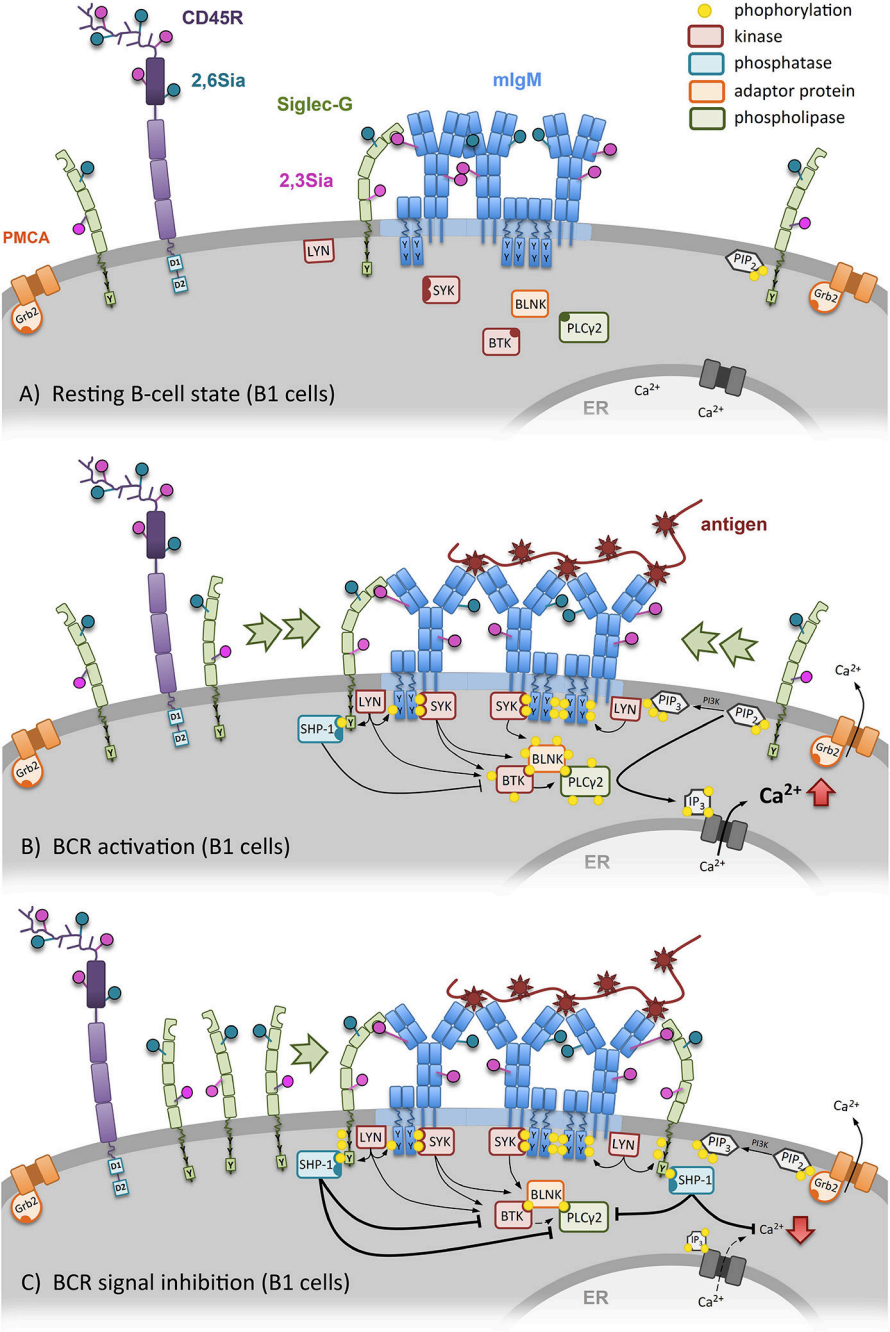
Siglec-G dependent regulation of the B-cell receptor (BCR) signal on B1 cells. **(A)** In resting B1 cells the conformation of the BCR is closed and already in steady-state Siglec-G directly binds to the Cμ1 domain of the BCR-IgM via its ligand-binding domain. **(B)** Specific antigen binding induces conformational opening and activation of the BCR, followed by Ca^2+^-mobilization. Subsequently, more Siglec-G molecules get recruited to the BCR. **(C)** Siglec-G recruitment inhibits BCR signaling. Siglec-G directly binds to the Cμ1 domain of the BCR-IgM via its ligand-binding domain. Due to vicinity of Siglec-G to BCR, the ITIM of Siglec-G gets phosphorylated by LYN, followed by SHP-1 binding and further inhibition of Ca^2+^ release.

The cellular function of Siglec-G was first demonstrated by analyzing Siglec-G deficient mice. These knockout mice revealed a functional importance of Siglec-G on the B1a-cell population, as this B-cell subset was extensively enlarged in Siglec-G deficient mice. Furthermore, the lack of this specific Siglec led to a higher calcium mobilization of B1a cells upon BCR stimulation (Figure 2). Interestingly B2 cells were not affected by the loss of Siglec-G, suggesting an inhibitory role of Siglec-G only on B1 cells (14, 31). This loss of regulation was assumed to be a result of defective SHP-1 recruitment to dampen BCR signaling. This conclusion was based on the data of human Siglec-10 and SHP-1 interaction (9), as well as SHP-1 deficient motheaten mice that resemble the B1a cell expansion of Siglec-G knockout mice (32, 33). A reasonable explanation of its functional specificity for the B1-cell subset can be the additional ligand binding of α2,3-linked sialic acids next to α2,6-linked sialic acids (17). This topic, however, will be discussed later in this review article in the section focusing on ligand binding function.

Another study observed that B1 cell survival and selection is regulated by Siglec-G. As already mentioned, loss of Siglec-G results in a B1-cell expansion (14). Jellusova et al. (34) observed in 2010 a prolonged life span of Siglec-G deficient B1a cells when injected into RAG^−/−^ mice as well as a reduced spontaneous apoptosis rate *in vitro*. Western blot analysis of purified peritoneal B1a cells revealed higher expression of transcription factors Bcl2 and NFATc1, providing a possible mechanism of lower apoptosis of Siglec-G deficient B1a cells. Furthermore, Siglec-G deficient B1a cells were reported to express a skewed BCR repertoire via less phosphatidylcholine (PtC)-binding capacity accompanied by a reduced usage of V_H_11 and V_H_12. Moreover, Siglec-G knockout mice showed more IgM specific antibodies for typical oxidation-specific epitopes, like oxidized LDL (34). Another interesting story focused in more detail on the role for Siglec-G in atherosclerosis (35). Siglec-G deficiency leads to reduced athereosclerosis in Ldlr knockout mice with less inflammation induced by oxidized LDL. This protective effect is mediated by the expanded B1-cell subset, which secrets more IgM recognizing oxidized LDL (35).

Giving the fact, that Siglec-G inhibits BCR signaling, some studies focused on the role of Siglec-G in preventing autoimmunity. Deficiencies of various inhibitory receptors (e.g., FcγRII2b, CD72, or PIR-B) have been reported to develop autoimmunity in mice (36–38). Moreover, the B cell specific deletion of the BCR-signaling inhibitory phosphatase SHP-1 (which phosphorylates Siglec-G) leads to systemic autoimmunity (33). As mentioned before, CD22 deficiency alone does not lead to an autoimmune disease on pure C57BL/6 background, demonstrating a redundant function for B-cell Siglecs. However, different studies regarding autoimmunity and Siglec-G were carried out. It has been observed that on a BALB/c background, the loss of Siglec-G cause an earlier onset and more severe progression of collagen-induced arthritis. The inflammation of knee joints, visualized via H&E staining, additionally revealed a higher histological arthritis score for Siglec-G knockout mice (20). In the same study the impact of Siglec-G in systemic lupus erythematosus (SLE) was analyzed using Siglec-G deficient mice backcrossed by speed congenics into the lupus-prone MRL/*lpr* lupus strain. MRL/*lpr* mice develop SLE, characterized by autoantibody development, lupus nephritis and an early death with 7–9 month of age (39). Loss of Siglec-G MRL/*lpr* mice exacerbated the progression of disease expressed by higher anti-dsDNA titers compared to standard MRL/*lpr* mice, indicating a more severe disease. In addition male mice that lack Siglec-G on this lupus prone background suffered earlier from kidney damage, while females had an earlier onset of proteinuria and lower survival rate compared to respective controls (20).

The data from Bökers et al. (20) showed for the first time an association between Siglec-G deficiency and autoimmunity. As these data were based on disease models, another study focused on age related development of spontaneous autoimmunity. Therefore, the previously described Siglec-G knockout mouse on BALB/c background (14), was backcrossed by speed congenics to C57BL/6. Measurement of autoantibodies, blood urea nitrogen (BUN), proteinuria, kidney damage and other parameters have been investigated, to determine the grade of age related spontaneous autoimmunity. Though BUN and proteinuria score appeared to be normal in Siglec-G deficient aging mice (70 weeks), immune complex deposition in the kidney accompanied by nonlethal kidney damage has been observed. Furthermore, these mice showed higher titers of autoantibodies. Also enhanced T-cell activation and elevated B-cell numbers, regarding germinal center B cells and plasma cells, have been detected (19). These data clearly indicate an important role of Siglec-G in maintaining tolerance and therefore preventing the development of autoreactive B cells.

## B cell siglecs – ligand binding determines signaling function

Within the last few years, the signaling field has strongly focused on plasma membrane organization of receptors on the B cell surface. New findings from different groups show that membrane receptors like IgM, IgD, or CD19 are not distributed randomly, but are organized in nanoscale clusters. After B-cell activation these are remodeled to initiate and amplify signaling in the cell (40, 41). This clearly shows how important organization, localization and clustering is to promote correct signaling within the B cell.

Also CD22 is organized in membrane clusters consisting of CD22 homooligomers that are distinct from the BCR. These formed homooligomers occur due to α2,6-linked sialic acids binding on neighboring CD22 molecules. This has originally been demonstrated by photo-crosslinking glycan ligands to CD22 (42). Recently determined structural data show a rod-like structure of CD22 on the B cell surface, making neighboring CD22 proteins carrying α2,6-linked sialic acids optimal accessible for these homooligomers (43). Upon BCR activation, CD22 is recruited to the BCR and is phosphorylated by Lyn, so the inhibitory signaling cascade is initiated (23). However, different groups asked the important question what happens to the B-cell signaling if the ligand binding function of CD22 is impaired? First results were obtained by analyzing ST6gal I knockout mice. These mice lack the sialyltransferase ST6gal I which is the main enzyme to produce Siaα2,6Gal linkages (44). Indeed, using Sambucus nigra lectin staining (a lectin which binds specific α2,6-linked sialic acids) and staining with a NeuGcα2,6Gal-PAA probe (ligand for murine CD22) Collins et al. revealed that in the ST6gal knockout mice CD22 is unmasked (45). This means CD22 is not binding to α2,6-linked sialic acids anymore and is not clustered in homooligomers. Interestingly this mouse shows a reduced BCR signaling with a lower calcium mobilization, indicating a weaker B-cell activation (46). As these mice have a general lack of α2,6-linked sialic acids, other groups aimed to analyse specifically the α2,6-linked sialic acid binding of CD22 in more detail. Therefore, two different mouse lines with mutations in the conserved ligand binding domain of CD22 were generated (24, 47). The results differ from each other, as Müller et al. (24) clearly showed a reduced BCR signaling with less calcium mobilization in their CD22-R130E knockin mouse, phenocopying the St6gal I knockout mouse. However, Poe et al. (47) did not see this hypoactivated phenotype. It is important to mention that this mouse additionally shows a lower CD22 and IgM expression and therefore the phenotypes of the different mutant mice should be compared with caution.

The studies of Müller et al. (24) used proximity ligation assays to demonstrate a higher association of CD22 with IgM in the resting state of the CD22-R130E mice compared to WT mice. This association was even enhanced upon BCR activation, therefore explaining the hyporeactive B-cell state. Further studies were conducted to analyse the CD22 mobility and organization in the plasma membrane. In resting B cells a total of 65,000 CD22 molecules are organized in nanoclusters with a density of 410 molecules/μm^2^ (48). Super resolution microscopy revealed that the CD22 nanoclusters are close but still apart to the BCR clusters. Additional dSTORM analysis, that have a localization precision of 10–30 nm (49, 50), show that CD22 nanoclusters come with a diameter of 100 nm. Making use of single particle tracking (51), Gasparrini et al. (48) nicely confirmed their *in silico* model and showed that a resting B cell has a median diffusion coefficient of 0.046 μm^2^/s for CD22. This high lateral mobility of CD22 leads to 90 % coverage of the B-cell plasma membrane with CD22 molecules within only 500 ms. In contrast, other molecules like IgM, IgD or CD19 appear to diffuse more slowly. Further studies of this group demonstrated that CD22 organization in the membrane is independent of cortical actin cytoskeleton, however the dynamics are. Like mentioned above, CD22 distribution on the cell surface seems to be highly dependent on its ability to bind α2,6-linked sialic acids. Gasparrini et al. (48) were able to draw similar conclusions as they observed smaller CD22 nanoclusters on CD22-R130E B cells using dSTORM analysis. In addition, they revealed a significant higher mobility of CD22 molecules that lack the α2,6-linked sialic acid binding site (48). As CD22-R130E B cells show hyporesponsiveness (24), a higher lateral mobility seems to be a good explanation for the stronger attenuated BCR signaling.

Another approach aiming to elucidate CD22 *cis*-binding partners was performed using a proximity labeling assay (52). Therefore, WT B cells or ST6Gal1^−/−^ B cells were labeled with HRP-conjugated anti-CD22, followed by biotin-tyramide incubation. As a result, all proteins in the vicinity of CD22 get biotinylated, which can be detected by anti-streptavidin western blot. In this study the authors claim that several proteins including CD22, IgM, and CD45 could be identified as α2,6-linked sialic-acid dependent *cis*-binding partners of CD22 (52), contradicting other studies with similar methods (53). However, this method can identify proteins in the vicinity of CD22, but cannot directly demonstrate sialic-acid dependent binding of ligands. Therefore, no statement can be made whether CD22 binds to IgM via α2,6-linked sialic acids. Genetic evidence from mouse models analysing CD22 ligand binding speaks against this model. It was clearly shown by proximity ligation assays that after mutation of CD22 ligand binding domain, the associated is even stronger to IgM, compared to WT controls (24). As a consequence of this, BCR signaling is stronger inhibited (24), which resembles the calcium mobilization assay of ST6Gal I deficient B cells (46). Also in St6Gal I knockout B cells a stronger CD22-IgM association was demonstrated (54).

**Figure 3:**
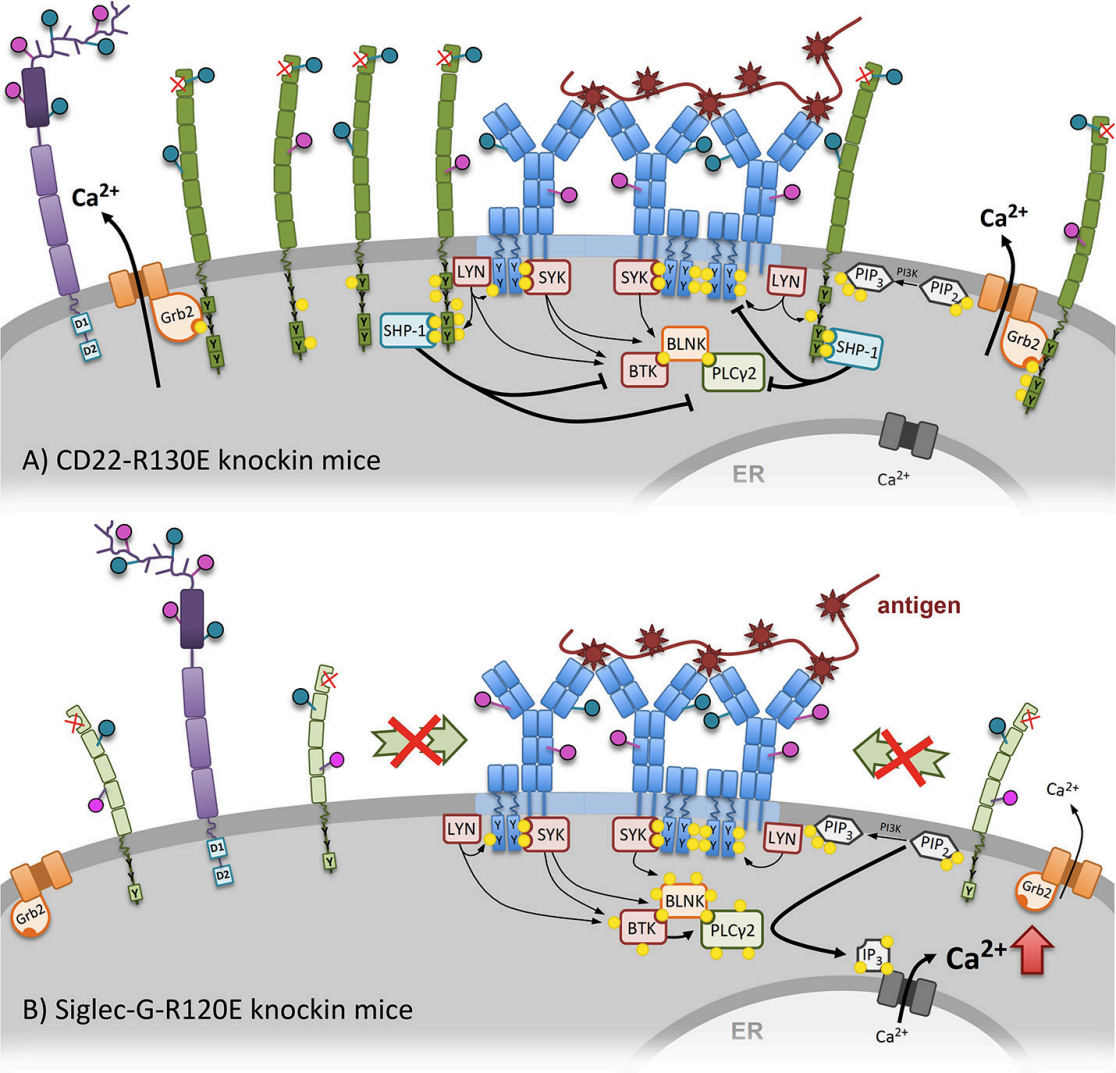
An altered calcium mobilization after mutation of the sialic-acid binding domains of CD22 and Siglec-G can be observed in CD22-R130E and Siglec-G-R120E knockin mice. **(A)** The mutated ligand-binding domain of CD22 of CD22-R130E knockin mice prevents CD22 homooligomers formation. Because of monomeric organization of CD22, it becomes more mobile at the cell surface. As a consequence more CD22 is associated with the BCR, gets stronger phosphorylated and can negatively intervene in the activation cascade of BCR signaling. The Ca^2+^ mobilization after BCR stimulation is strongly inhibited in these mice compared to wildtype. **(B)** In contrast, mutation of the ligand-binding domain of Siglec-G leads to elevated Ca^2+^ mobilization after BCR stimulation in B1 cells. Recruitment of Siglec-G to the BCR is dependent on direct sialic acid binding and prevented in the case of Siglec-G-R120E mice. Already in steady state on association of Siglec-G with the BCR can be detected.

Based on the discussed data the current view of how CD22 ligand binding affects BCR inhibition was generated. In the resting B cell (Figure 1A), CD22 is clustered in homooligomers (by α2,6-linked sialic acid binding in *cis*) apart from the BCR. Upon BCR stimulation (Figure 1B) the homooligomers get recruited to the BCR, CD22 is phosphorylated and can promote its inhibiting function (Figure 1C). Furthermore, *trans* and *cis* ligands of CD22 compete with each other. However, if the ligand binding function of CD22 is disturbed, CD22 is organized in smaller clusters in the plasma membrane, which show a higher mobility on the B-cell surface. Therefore, more CD22 can faster interact with the BCR to inhibit signaling (Figure 3A).

As mentioned in the beginning, CD22 is not the only known Siglec on B cells. Siglec-G, which is not so well-studied as CD22, but deserves to be noted as well. A study from the year 2014 concentrated on the role of Siglec-G ligand binding function by generating a Siglec-G-R120E knockin mouse. The mutated Siglec-G lacks the ability to bind its ligands α2,3- and α2,6-linked sialic acid (4). Interestingly, these mice do not phenocopy the hyporeactive CD22-R130E mouse with lower calcium mobilization and a hyporeactive state, mentioned above (24). Instead, they resemble the phenotype of the total Siglec-G knockout, which is characterized by enhanced calcium signaling in B1a cells and massive expansion of this subpopulation (14). Proximity ligation assay of the Siglec-G-R120E mouse revealed that already in the steady state and also upon BCR activation the association of Siglec-G with IgM is strongly disturbed. This shows that *cis* ligand binding is important for the inhibitory function of Siglec-G. However, in contrast of CD22, the loss of the ligand binding abolishes the inhibitory function on B1a cells and leads to a hyperactive state (4). Siglec-G seems to bind directly to sialic acids coupled to the cμ1 domain of surface IgM (Özgör et al., unpublished observations). The impact of Siglec-G ligand binding function is summarized in Figure 3B. The B1a cell restricted phenotype is explained by the fact that Siglec-G has a different sialic acid binding pattern, as it recognizes also α2,3-linked sialic acids besides α2,6 linkages (17). Interestingly more α2,3-linked sialic acids are expressed on B1 cells compared to B2 cells (4). This clearly shows, that the inhibitory function of both Siglecs is not only differentially regulated through ligand binding specificity, but also dependent on the respective B-cell subset.

Next to *cis*-binding of CD22 of sialic acids on the same cell, CD22 is capable to bind its ligand also on opposing cells in *trans* (2). *Cis*-binding is supposed to limit the association of CD22 with the BCR by forming homooligomeres (24, 42), whereas *trans*-binding is thought to redistribute CD22 and IgM to the site of cell contact and can suppress B-cell activation (18, 55). Moreover, Macauley et al. (56) could provide an additional potential of *trans*-interaction. Tolerance was induced by using liposomes, displaying not only protein antigens, but also high-affinity CD22-ligands, resulting in the depletion of antigen-reactive B cells via apoptosis. Natural *cis*-ligands on the cell surface are “masking” CD22 for *trans*-ligands (57), thereby setting a competitive threshold to regulate *trans*-binding (58). Some studies were focusing on soluble *trans*-ligands, which are capable to overcome *cis*-binding of CD22. Courtney et al. (59) used bifunctional sialylated antigens, which interact with the BCR and CD22, causing an initial BCR signal, followed by suppression of downstream effectors through CD22 (59). CD22 *trans*-recognition of α2,6-linked sialic acids may also play an important role in abolishing autoreactivity. Therefore, Lanoue et al. (60) transfected target cells to produce the CD22-ligand and could show a suppression of BCR signaling in vitro, probably due to CD22 recruitment via *trans*-binding. Two further studies could verify an induction of tolerance *in vivo* by encounter of B cells with specific antigens on cell surfaces or liposomes decorated with ligands for CD22 (17, 61). The immune response of antigen-specific B cells was abolished, even if the cells were restimulated with the unsialylated form of the carrier (17). Based on these findings, the regulatory role of CD22 is dependent on both, *trans* and *cis* interactions (Figure 1).

As knockout mice, deficient for both inhibitory Co-receptors, CD22 and Siglec-G, show a progressive autoimmune disease upon aging with lupus-like symptoms (21), the question arose whether the ligand binding function of both Siglecs is important to prevent autoimmunity. Sialic acids are self-associated patterns and therefore it has been postulated that Siglec binding to sialic acids (*cis* or *trans*) may induce tolerogenic signals (62–64). A quite recent study investigated this issue by using Siglec-G R120E × CD22-R130E mice. These animals express mutated Siglec-G and CD22, which leads to a loss of ligand binding function for both Siglecs (65). The authors observed that the opposing phenotype of the CD22-R130E and Siglec-G-R120E mice seems to be mostly restricted to the respective B-cell subset that is regulated by CD22 or Siglec-G. Furthermore, these mice did not develop age-related autoimmunity, measured by BUN, proteinuria, autoantibodies and flow cytometry analysis of B- and T-cell subsets. This showed that ligand binding of CD22 and Siglec-G can not be a dominant mechanism for B cell tolerance induction (65).

## CD22 & CD45 – update on their relationship

For some time, CD45, a highly glycosylated protein (66, 67), has been handled as a putative binding partner for CD22 (53, 68–70). In the last years it has gained again some interest as Coughlin et al. (71) had a closer look at the function of the extracellular domain of CD45. Besides the well-studied cytoplasmic tail with tyrosine phosphatase activity, the role of the extracellular domain (also referred to as extracatalytic) has been stayed elusive. To finally solve this function, Coughlin et al. (71) used different knockout and transgenic mice, to dissect extracatalytic from phosphatase activity. By providing genetic evidence, they showed an interaction between CD45 and CD22 in the steady state (71). This association is mediated through the extracatalytic function of the extracellular domain of CD45. The authors propose, that the interaction of CD45 and CD22 might be dependent on *cis* sialic acid binding, but this has not been directly shown. Furthermore, they suggest that the interaction between CD22 and CD45 restrains CD22 from inhibiting tonic BCR signaling, as mutant CD45 mice without catalytic activity show reduced basal calcium signaling. Indeed, data from mutant mice, lacking the extracatalytic function of CD45 partially resemble the phenotype of CD22-R130E mice. The authors conclude that the interaction between CD45 and CD22 limits association of CD22 to the BCR in resting B cells (71). Further, evidence for a sialic acid based interaction of those two molecules comes from super resolution microscopy studies. The group of Batista studied the organization of CD22 in the plasma membrane in CD45 knockout mice (48). In this mouse strain, they found a drastically altered CD22 distribution revealing larger CD22 nanoclusters accompanied by a lower diffusion coefficient. This effect was observed to be CD45 dose dependent, since analyses of CD45^+/−^ heterozygous mice show CD22 clustering proportionally dependent on the amount of CD45 on the cell surface. Further experiments, using CD45 knockout cells, treated with sialidase to remove sialic acids, demonstrated that the CD22 nanocluster were again smaller. With respect to older data showing CD22 homooligomer clustering (42) they reasoned that upon removal of sialylated CD45, the competition for CD22 *cis* binding partners is reduced. As a consequence CD22 clusters more in homooligomers, which in turn are bigger in size (48).

## Galectin-9 – immunomodulatory effects on B cells

Besides sialic acids, a huge variety of other glycans exist on mammalian cells (72) and the B cell glycome has only been described in detail 10 years ago (73). Galectin-9 (Gal-9) is a member of the S-type lectin family, the galectins, known to bind a broad range of N-acetyllactosamine-containing glycans (74). Gal-9 itself has two carbohydrate-recognition domains and can therefore bind several selected N-glycans and repeated oligolactosamines (75). Binding of galectins can have immunomodulatory effects on the respective cell (75–80) and galectin-glycoprotein interaction can influence membrane protein organization and mobility by forming glycan-based domains (81–84). Interestingly, Gal-9 knockout mice show a B cell phenotype with higher proliferation and larger GC (78, 80). Two quite recent studies focused on the role of Gal-9 on B cells with respect to BCR signaling and membrane organization and found interesting new connections to IgM, CD45 and CD22.

N-glycan mass spectrometry analysis was used to investigate N-glycan structures on surface glycoproteins of naïve, germinal center (GC) and memory B cells (B_mem_), sorted from human tonsils (85). They revealed that poly-N-acteyllactosamine (poly-LacNAc) was highly abundant on all three B-cell subsets, however, structural differences appeared between the GC and the two other groups. While poly-LacNAc glycans of naïve and B_mem_ are build linearly, GC B cells characteristically showed poly-LacNAc modifications known as I-branches (85). Further, analyses revealed reduced Gal-9 binding only to GC B cells, presumably caused by I-branching of poly-LacNAc. This was verified by characterizing human B-cell lines (either derived from GC or non GC cells) that have been transfected with β1,6-N-acetylglucosaminyltransferase (GCTN2) knockdown or overexpressing constructs. GCTN2 is a glycosyltransferase with presumably exclusive I-branching activity on N-glycans (86, 87), and is therefore the enzyme mediating the changes in poly-LacNAc structures from linear on naïve B cells to I-branched on GC B cells. Results confirmed first assumptions that I-branched poly-LacNAc is hardly bound by Gal-9 (85). In contrast to Giovannone et al. (85), another work focused on murine B cells and was able to confirm Gal-9 binding as well (88). In both studies (85, 88), CD45 was detected as binding partner of Gal-9, using different (co-) immunoprecipitation or pull-down approaches with recombinant Gal-9, while Cao et al. additionally detected IgM as Gal-9 interacting protein. Whereas, Cao et al. (88) focused mainly on Gal-9 influence on membrane organization of CD45 and IgM, Giovannone et al. (85) analyzed signal transduction in more detail. A Gal-9 dose-dependent (range of 0–2 μg/ml Gal-9) induction of tyrosine phosphorylation of Lyn, CD22 and SHP-1 was a first hint for Gal-9 suppressing BCR signaling cascade. Indeed they confirmed this idea by using calcium mobilization assays on human tonsil B cells, showing that Gal-9 treatment together with anti-IgM stimulation led to a striking reduction of calcium influx (85). As mentioned before, CD22 and CD45 extracellular domain have been suggested to interact together via sialic acid binding in resting B cells (48, 71). To investigate whether the Gal-9 immunomodulatory effect is dependent on protein clustering introduced by sialic acid binding, experiments after sialidase treatment have been conducted. Removal of sialic acids abrogated the Gal-9 inhibitory effects on naïve B cells, shown by calcium measurement. Of course one has to mention that sialidase treatment alone dampens BCR signaling, as a consequence of breaking up *cis*-interactions of CD22 (24). However, because adding Gal-9 after removal of sialic acids did not lead to a stronger inhibition, the authors suggest that Gal-9 binding to CD45 induces a CD22-dependent immunomodulatory effect through the Lyn-CD22-SHP-1 pathway in order to inhibit BCR signaling and activation.

Murine Gal-9 knockout B cells show enhanced IgM-BCR microcluster formation after application to anti-kappa-fluorochrome coated lipid bilayers (89), furthermore increased total tyrosine phosphorylation, and especially more phosphorylated ERK1/2, could be observed (88). Interestingly, adding 0.1 μM rGal-9 to the knockout cells could rescue this phenotype, showing an impact of Gal-9 on BCR signaling. Further experiments of this group, focused on the role of Gal-9 with respect to membrane organization (88). Therefore, dSTORM microscopy was applied, known to reach a lateral resolution of approximately 20 nm (49, 50). Analysis showed no differences in nanoscale IgM clustering or clustering tendency between murine Gal-9 deficient and WT B cells, leading to the suggestion that Gal-9 is not involved in forming IgM nanoscale cluster. However, treating Gal-9 deficient cells with rGal9 resulted in a reduction of IgM cluster numbers, which in turn were bigger in size with an icreased number of molecules. To investigate its influence on IgM mobility, single particle tracking of IgM was performed. Thereby they observed that Gal-9 deficient B cells have an 30% higher median diffusion coefficient, meaning IgM moves faster in cells lacking Gal-9. Interestingly, incubating cells with fluorochrome conjugated rGal-9 led to an unequal distribution to one side of the cell forming a cap and therefore giving rise to Gal-9^high^ and Gal-9^low^ areas. Comparing these two areas showed that Gal-9 binding enhances not only CD45, but also CD22 and CD19 density within the Gal-9-glycoprotein lattice. Further dSTORM experiments showed that CD22 and IgM co-localization is reduced in Gal-9 knockout cells, giving a possible explanation for the enhanced BCR signaling in Gal-9 deficient B cells. To conclude the authors propose, Gal-9 is important for merging pre-existing IgM nanoclusters, its immobilization and the relocalisation of IgM together with CD45 and CD22 (88).

## CD22 beyond naÏve B cells – function on germinal center and memory B cells

Most of the conducted CD22 studies focused on solving its signaling pathway and ligand binding in naive B cells. In 2015 a work from Macauley et al. (90) was published concentrating on CD22 ligand binding during the GC reaction. They showed, that the expression of the high affinity ligand of CD22 is selectively downregulated only during GC B-cell stage, whereas on memory B cells it is upregulated again (90). Therefore, CD22 is unmasked on GC B cells, meaning no binding of its ligand in *cis* is possible. Even though the high affine CD22 ligands differ from mice to man (in mouse Neu5Gc and in human sulfonated Neu5Ac-containing glycan), and the mechanism of restricting high affinity ligand access in GC B cells is different, both species show the same pattern of ligand downregulation on GC B cells and upregulation again during memory B-cell stage. The authors did not look into the functional meaning of this unmasking process of CD22, however they give several possible explanations. One idea is that the enhanced *trans* binding possibility of CD22 on GC B cells might provide some kind of checkpoint for B cells that acquired autoreactivity during the GC reaction. This idea is based on previous work from the same group focusing on *trans* ligand binding. They showed that autoantigen expressing cells recruit CD22 to immunological synapse via *trans* ligands (62). Other ideas suggest a role in dark zone to light zone migration or involvement in T-B-cell interaction or a role in binding of sialylated IgG immune complexes (90).

First evidence for a role of CD22 in homing processes has been published in 2014, revealing that B cell homing to intestinal lymphoid tissue is dependent on α2,6-linked sialic acids (91). They showed that compared to high-endothelial venules (HEV) of peripheral lymph nodes (PLN), ST6gal I, the enzyme involved in generating Siaα2,6Gal linkages, is particularly expressed in HEVs of mesenteric lymph nodes (MLN) and to an even higher extent in Peyer's patches' (PP) HEVs. As α2,6-linked sialic acids are ligands to CD22, and the observation that B cell homing to PP is more effective than to MLN, encouraged further analysis. Indeed, short term homing assays of CD22 knockout and WT cells injected into WT controls, revealed a reduced homing of CD22 deficient B cells exclusively to PP and MLN. Repeating these experiments with ST6gal I knockout recipients, showed reduced homing to MLN and PP of both B cell genotypes, indicating an α2,6-linked sialic acid dependency (91). These data highlight a role for CD22 and its ligand binding in lymphoid organ homing, leaving ideas for speculation on detailed mechanisms and other possible migration functions, like for example the inter-zone movement in germinal centers, as mentioned by Macauley et al. (90).

Going further to the memory B-cell stage, a recent publication focused on the role of CD22 for memory B-cell formation and revealed a requirement for CD22 expression to form memory B-cell precursor within the GC (92). These studies show that there is still a lot to discover about the role of CD22 beyond the naïve B cell.

## Human CD22 – from bench to bedside

Members of the Siglec family have some features making them attractive targets for immunotherapy. These characteristics are the restricted expression pattern, the rapid endocytosis upon engagement with antibodies and the ability to modulate cellular signaling (93). As CD22 expression is restricted to B cells it can serve as important target for immunotherapy of B cell mediated autoimmune diseases and B cell related lymphomas. B cells can contribute to the pathogenesis of autoimmune diseases, for example in SLE, B cells produce autoantibodies leading to the deposition of immune complexes in several organs (94, 95). Therefore, therapeutic approaches to either eliminate autoreactive B cells or to induce tolerance are needed.

Therapeutic antibodies against cell specific targets can be used for immunotherapy. One antibody targeting CD22 is epratuzumab. In contrast to the anti-CD20 antibody rituximab, it induces no complement-dependent cytotoxicity (CDC) and only moderate antibody-dependent cellular cytotoxicity (ADCC) (96). However, it rather modulates BCR signaling like calcium mobilization and phosphorylation of downstream signaling molecules (97, 98). Epratuzumab has been used in clinical trials with systemic lupus erythematosus (SLE) patients but finally failed in the phase III study (99). However, recently it could be shown that patients with SLE and associated Sjögren's syndrome treated with epratuzumab showed improvement in SLE disease activity (100).

Another new approach to induce antigen-specific suppression and to treat autoimmunity in the future is the usage of liposomal nanoparticles that display antigen and glycan ligands for the inhibitory receptor CD22. These so called STALs (SIGLEC-engaging tolerance-inducing antigenic liposomes) induce antigen-specific tolerance and selectively induce apoptosis (56).

CD22 is not only expressed on healthy or autoreactive B cells but also on the majority of B-cell lymphomas (101) and on 65% of the acute lymphoblastic leukemia (ALL) (102). CD22 is known to be an internalizing surface receptor (103, 104). Therefore antibodies, antibody-based or sialoside-based immunotoxins can be used to target the cell via CD22. Moxetumomab pasudotox, an antibody-based anti-CD22 immunotoxin showed evidence of activity in relapsed or refractory childhood ALL during phase I study (105). In addition, it offers a clinically meaningful treatment for patients with hairy cell leukemia (106). The antibody-drug conjugate inotuzumab ozogamicin, an anti-CD22 antibody coupled to the cytotoxic antibiotic calicheamicin, demonstrated superior clinical activity for relapsed/refractory B-cell ALL (107). To increase targeting specificity, bispecific antibodies can be used. A bispecific antibody (DT2219) targeting CD22 and CD19 has been coupled to diphtheria toxin and preliminary clinical activity could be observed in a Phase I study with patients suffering from relapsed/refractory lymphoma or leukemia (108).

However, therapeutic antibodies or antibody-based therapeutics can lead to side effects, for example due to binding of the complement or Fc receptors. Furthermore, these antibodies are usually expensive therapies. Therefore, targeting Siglecs with their glycan ligands could represent another approach with lower immunogenicity (93, 109). Synthetic sialic acid-containing glycans can also be used to target CD22. It is important that these ligands have a high affinity and are capable to outcompete the endogenous *cis* ligands of CD22 (58). Like antibody-based immunotoxins these CD22 ligands can be used to deliver toxins into a target cell. As an example, Pseudomonas exotoxin A has been coupled to synthetic sialosides, newly developed high affinity ligands of human CD22. These constructs specifically kill CD22-positive B-cell lymphoma cells *in vitro* (110). Furthermore, high affinity ligands, loaded with doxorubicin and conjugated to the surface of liposomal nanoparticles, induced rapid endocytosis and killing of B cell lymphoma cells (111). Recently, it could be shown that CD22 ligands based on a di- or trivalent N-glycan scaffold had up to 1500-fold increased affinity, compared to the monovalent ligands. Conjugates of these multivalent ligands with auristatin and saporin toxins were internalized and killed the B cell lymphoma cells (112).

CAR T cell therapy has drastically altered the field of treatment of leukemia. CD19-directed CAR T cells showed promising results and have recently been approved by the FDA for the use in children and young adults ALL and in adults with large B cell lymphoma (113). Nevertheless, further therapies need to be developed as anti-CD19 therapy can fail due to the expression of truncated CD19 variants or the loss of CD19 (114, 115). Therefore, CD22-targeting CARs should also be considered. CAR T cells express an extracellular antibody fragment recognizing the tumor antigen together with a transmembrane domain and intracellular T cell signaling domain. These cells are generated by transduction of T cells from the patient and induce MHC-independent lysis of the tumor cell expressing the target antigen (116). Anti-leukemic activity of CD22 CAR T cells could be shown *in vitro* and *in vivo* by mouse experiments (117). First results from a phase I trial also showed clinical activity of CD22 CAR T cells for the treatment of B-ALL patients. These study included patients that were resistant to anti-CD19 immunotherapy highlighting the importance of CD22 CAR T cells in addition to CD19 CAR T cells (118).

Recently the molecular structure of the extracellular portion of human CD22 has been solved including the target site of epratuzumab. These data indicate that the CD22 glycosylation impacts the ability of the therapeutic antibody to access its epitope. The structural insights now enable the structure-based development of new therapeutic reagents (43). For better understanding of *in vivo* effects of CD22-based immunotherapy a transgenic mouse expressing human CD22 can be beneficial. Two different knockin mice were generated which express human instead of murine CD22 on the B-cell surface (119, 120). These animals can now be used to investigate the mode of action and side effects of anti-human CD22 therapeutic antibodies, immunotoxins or CAR T cells and to further optimize therapies of B-cell malignancies and autoimmunity.

In conclusion, CD22 and Siglec-G are important inhibitory receptors on B cells that control the BCR signaling threshold, preventing a too strong B cell activation that may lead to autoimmune disease induction. The inhibitory functions of these two Siglecs are tightly regulated by ligand interactions, which determine their organization in membrane microdomains and their vicinity to the BCR. The role of the relative contribution of *cis* and *trans* ligands in this regulation is still an open question. There are interesting changes occurring in the sialic acid ligand expression on B cells during germinal center responses, which are so far not understood mechanistically. Furthermore, CD22 has been shown to be a promising target in autoimmune diseases and B cell leukemias and it is expected that Siglec-10 will follow as a target in the future.

## Author contributions

SM, AL, CB, and LN wrote the manuscript. LN supervised the writing and submission.

### Conflict of interest statement

The authors declare that the research was conducted in the absence of any commercial or financial relationships that could be construed as a potential conflict of interest.
